# Spin and Methodological Quality of Systematic Reviews and Meta‐Analyses Comparing Single‐ and Multiple‐Visit Endodontic Treatment: A Meta‐Research Study

**DOI:** 10.1111/aej.70087

**Published:** 2026-05-22

**Authors:** Ana Luiza Cardoso Pires, Laylla Galdino‐Santos, Bas Loomans, Tatiana Pereira‐Cenci, Luiz Alexandre Chisini, Fernanda Geraldo Pappen

**Affiliations:** ^1^ Federal University of Pelotas Pelotas RS Brazil; ^2^ Department of Dentistry Research Institute for Medical Innovation, Radboud University Medical Center Nijmegen the Netherlands

## Abstract

This study evaluated the methodological quality and presence of spin in systematic reviews with meta‐analyses comparing single‐visit and multiple‐visit endodontic treatment, and investigated factors associated. Systematic reviews of randomised clinical trials were included without restrictions on language or publication year. Narrative reviews, reviews without meta‐analysis, non‐randomised or non‐clinical studies, and conference abstracts were excluded. A comprehensive search was conducted in PubMed, Scopus, Web of Science, the Cochrane Library and grey literature in January 2025. Spin was classified using the Yavchitz et al. framework, and methodological quality was assessed with AMSTAR 2. Data extraction and spin assessment were performed independently and in duplicate. Thirteen systematic reviews were included. Spin was identified in 12 studies of abstracts and 10 of full texts, mainly as a misleading interpretation. Most reviews were rated as low or critically low quality. Positive results and absence of risk‐of‐bias assessment were associated with increased spin in full texts.

## 1 Introduction

The choice between single‐ and multiple‐visit root canal treatment remains a relevant and extensively debated issue in dental practice [1]. The single‐visit protocol offers several advantages, including fewer appointments, reduced overall treatment time and cost, and greater convenience for patients [1, 2]. Conversely, the multiple‐visit approach may be preferable in certain clinical situations, such as cases involving persistent canal moisture, limited chair time or the need for stricter infection control [3].

The main distinction lies in the use of intracanal medicaments, most commonly calcium hydroxide, between appointments in the multiple‐visit approach [4]. In contrast, in single‐visit protocols, all stages, including instrumentation, disinfection and obturation, are completed in a single session [2]. Although extensively investigated, current evidence has not established the clinical superiority of either approach [5]. Available studies suggest that these procedural variations do not result in meaningful differences in outcomes such as treatment success, periapical healing or postoperative pain, regardless of pulpal or periapical status [6, 7].

Systematic reviews play a critical role in synthesising scientific evidence and guiding clinical decision‐making [8]. However, the interpretation of their findings may be compromised by methodological bias or by the intentional or unintentional distortion of results through narrative manipulation, a practice known as ‘spin’ [9, 10]. Spin refers to reporting strategies that present results in a biased manner, often by overstating favourable outcomes or downplaying unfavourable ones, even when the data do not fully support such interpretations [11]. This practice is often subtle and may reflect academic or editorial pressures rather than deliberate misconduct [9].

An example of spin that can directly influence clinical decision‐making is when authors highlight a statistically significant difference in a secondary outcome, such as short‐term postoperative pain reduction, as if it demonstrated the overall superiority of a single‐visit endodontic treatment, despite the absence of differences in the primary outcome (e.g., long‐term periapical healing or treatment success). Selective emphasis on specific endpoints may mislead clinicians and readers, ultimately influencing both scientific interpretation and clinical judgement. Although research should be reported transparently and accurately, meta‐research studies have increasingly identified such distortions in the biomedical literature [12, 13]. Recognising and addressing these practices is crucial to safeguarding research integrity and supporting evidence‐based clinical care [10].

Given the lack of consensus in the literature regarding the superiority of single‐visit versus multiple‐visit endodontic treatment protocols, and considering the potential influence of methodological and narrative biases on the outcomes of systematic reviews, it becomes essential to evaluate these publications critically. Although spin has been previously investigated in abstracts of systematic reviews in Endodontics [14], no study to date has comprehensively evaluated spin in both abstracts and full texts of systematic reviews with meta‐analyses while also assessing methodological quality and factors associated with its occurrence. Therefore, the present meta‐research aims to fill this gap by systematically evaluating the prevalence, severity and determinants of spin in systematic reviews comparing single‐visit and multiple‐visit endodontic treatments.

## 2 Materials and Methods

### 2.1 Study Design and Setting

The reporting of this meta‐research study followed the PRISMA guidelines [15] (Supporting Information S1) and, where applicable, the reporting recommendations for meta‐research studies proposed by Murad and Wang [16]. Currently, no specific reporting guideline has been developed exclusively for meta‐research; therefore, PRISMA remains the most widely adopted framework for this type of investigation. The study protocol is publicly available in the Open Science Framework (OSF) repository (https://osf.io/wxnp8/). Amendments were made after registration to include two additional authors (L.A.C. and B.L.), who contributed substantially to study design, statistical analysis and manuscript preparation.

### 2.2 Eligibility Criteria

Systematic reviews with meta‐analyses of randomised clinical trials in the field of endodontics comparing single‐visit versus multiple‐visit root canal treatment were included in this study. No restrictions were applied regarding year of publication or language. The following types of publications were excluded: brief communications, letters to the editor, narrative reviews, systematic reviews without meta‐analyses, in vitro studies, animal studies, conference abstracts, case reports and case series, technical reports, case–control studies, qualitative studies or any other type of study design not meeting the inclusion criteria.

### 2.3 Literature Search and Study Selection

A comprehensive literature search was conducted in the following electronic databases: PubMed MEDLINE, Scopus, Web of Science and the Cochrane Collaboration Library. Additionally, a manual search was performed in the IADR Abstract Archives (grey literature). The search strategy was conducted in January 2025, using MeSH terms and other relevant keywords, with adjustments made for each database (Supporting Information S2). All records retrieved were first imported into EndNote X7 reference management software (Thomson Reuters, New York, NY, USA) for duplicate removal. Subsequently, two independent reviewers (A.L.C.P. and T.P.C.) screened the titles and abstracts in duplicate and in a blinded manner using the Rayyan web application [17] to identify potentially eligible articles.

Two independent reviewers screened titles and abstracts in duplicate. Inter‐reviewer agreement for title and abstract screening (*κ* = 0.79) and for full‐text assessment (*κ* = 0.81). Disagreements were resolved through discussion and consensus. A list of excluded studies, along with the reasons for exclusion, was documented (Supporting Information S3).

### 2.4 Data Extraction

The following variables were extracted:

- Journal‐related data: journal name, metrics (CiteScore and impact factor), abstract word count, endorsement of ICMJE (International Committee of Medical Journal Editors) and PRISMA guidelines (yes/no), and open access status (yes/no).
- Article‐related data: first author, year of publication, country and continent (based on the first author's affiliation), article title, compliance with the journal's abstract word limit (yes/no), number of primary and secondary outcomes, distinction between primary and secondary outcomes (yes/no, as stated in the full text), use of structured abstract (yes/no), declaration of adherence to PRISMA guidelines (yes/no, mentioned in the full text), statement of funding source (yes/no), conflict of interest disclosure (yes/no), protocol registration status (yes/no), registration number, type of registration (prospective or retrospective, with prospective defined as registration prior to study initiation), risk of bias assessment (yes/no), risk of bias assessment tool used, use of GRADE (Grading of Recommendations Assessment, Development and Evaluation) to evaluate the certainty of evidence (yes/no), citation rate per year, number of citations in Scopus, number of primary studies included in the meta‐analysis, presence of spin in the abstract and full text, and categorization of the primary outcome results of the included studies (including whether the primary outcome was clearly defined, the number of primary and secondary outcomes, and whether the outcome was statistically significant, positive or negative).

Data were extracted and coded in duplicate using Microsoft Excel (Microsoft Corp., Washington, USA) by two independent reviewers (A.L.C.P. and L.G.S.), taking into account the subjective nature of identifying spin strategies. In cases of disagreement, the articles were thoroughly reviewed and discussed with a third reviewer (T.P.‐C.) until a final consensus was reached.

### 2.5 Covariates

A set of covariates, pre‐defined based on their relevance for explaining heterogeneity in the presence and intensity of spin, was used in the statistical analyses [12, 17, 18, 19, 20, 21]. These variables encompassed bibliometric characteristics (year of publication, continent, open access status), methodological elements (specification of primary and secondary outcomes, PRISMA citation, risk of bias assessment, use of GRADE, protocol registration) and reporting aspects (compliance with abstract word limit, abstract format (structured vs. unstructured), funding source, conflict of interest disclosure, direction of outcome results). Additional data were extracted to characterise the sample but were not included in the statistical models. The complete list of extracted items is available in Supporting Information S4.

### 2.6 Spin Classification

#### 2.6.1 Definition and Operationalisation of Spin Outcomes

The phenomenon of spin was evaluated through three distinct outcomes, each capturing a different dimension of reporting bias:

1. Spin Frequency in Abstracts: A discrete count variable representing the total number of distinct spins identified in the abstract, based on a 21‐item checklist. The theoretical range is 0 to 21.
2. Spin Severity in Abstracts: An ordinal categorical variable classifying abstracts into three mutually exclusive tiers of methodological distortion:
  - None: No spin elements were identified (count = 0).
  - Low‐level: One or more spins were present, but none belonged to the pre‐specified list of nine most severe forms of spin.
  - High‐level: At least one of the nine most severe spin elements was identified.
3. Spin Frequency in Full Texts: A discrete count variable representing the total number of distinct spins identified in the main body of the review, based on a comprehensive 28‐item checklist. The theoretical range is 0 to 28.

#### 2.6.2 Measurement Instrument and Spin Typology

The assessment of spin was conducted using the classification framework developed by Yavchitz et al. [22]. This framework was developed through expert consensus and has been widely used in meta‐research to identify spin in systematic reviews and randomised clinical trials. This instrument has been previously applied in multiple dental specialties [12, 23, 24, 25, 26], supporting its validity and reproducibility in dental research.

The criteria categorise spin into three domains: misleading reporting, misleading interpretation and inappropriate extrapolation.

- Misleading Reporting: Pertains to omissions or inadequate descriptions in the presentation of the review's methodology, analysis or results. This domain contains 8 items for abstract assessment and 10 for full‐text assessment.
- Misleading Interpretation: Concerns the framing and discussion of results in a way that distorts their meaning. This is the largest domain, comprising 10 items for abstracts and 13 for full texts.
- Inappropriate Extrapolation: Involves unjustified generalisations that extend the findings beyond the scope of the available evidence. This domain includes three items for abstracts and five for full texts.

The complete list of all 21 abstract‐specific and 28 full‐text‐specific criteria, including verbatim examples, is provided in Supporting Information S5. Spin identification was conducted independently and in duplicate. The initial inter‐reviewer agreement was *k* = 0.70. A calibration meeting was held to standardise the interpretation of the spin criteria, which resulted in a value of agreement *κ* = 0.94. Disagreements were resolved through discussion and consensus, and a third reviewer was consulted when necessary.

#### 2.6.3 Considerations for the Ordinal Outcome

High‐level spin was defined as the presence of at least one of the nine most severe spin elements. Low‐level spin was assigned when none of these nine severe spin elements were identified. The complete absence of any spin elements, regardless of severity, was classified as no spin (Supporting Information S6).

### 2.7 Methodological Quality

The methodological quality of each systematic review was assessed using the AMSTAR 2 tool [27, 28]. Each review received a rating of high, moderate, low or critically low according to its performance across multiple domains, as defined by the online platform (https://amstar.ca/Amstar_Checklist.php). Data extraction and quality assessment were conducted independently by two reviewers (A.L.C.P. and L.G.S.). Any discrepancies were resolved through discussion and consensus, and a third reviewer (T.P.‐C.) was consulted when necessary to reach a final decision.

### 2.8 Data Analysis

Quantitative variables were described using means and standard deviations, while qualitative variables were presented as absolute and relative frequencies. To assess associations between study characteristics and the presence of spin in abstracts and full texts, Student's *t*‐test or one‐way ANOVA were used, as appropriate.

Poisson regression models with robust variance were used to estimate rate ratios (RR) and their corresponding 95% confidence intervals (95% CI) for the number of spin occurrences identified in the full texts of systematic reviews. However, given the limited sample size of the study (*n* = 13), these regression models were conducted only as exploratory analyses. To avoid overinterpretation of potentially unstable estimates, the results of these analyses are presented only in the Supporting Information.

The level of spin in abstracts was initially classified as an ordinal variable with three categories (none, low, high). However, the distribution of this variable was highly skewed, with only one (7.7%) of the 13 included studies categorised as having ‘no spin’. This low frequency in the reference category, combined with the limited sample size, resulted in a rare‐event scenario that led to quasi‐complete separation and biased maximum likelihood estimates when using conventional ordinal logistic regression [29].

To address this limitation and obtain more stable estimates, Firth's penalised likelihood logistic regression was applied [30]. This method is specifically designed for small sample sizes and rare events, providing less biased estimates in situations involving data separation. For this analysis, the outcome variable was dichotomised as ‘any spin’ versus ‘no spin’, preserving the distinction between systematic reviews completely free of spin and those presenting any degree of distorted reporting or interpretation.

Odds ratios (ORs) and their corresponding 95% confidence intervals (95% CIs) were estimated using the profile penalised log‐likelihood method. Regression analyses were conducted for exploratory purposes only. Covariates were initially considered for inclusion in the multivariable model through a backward stepwise selection procedure. Variables with a *p* value < 0.250 in the univariable analysis were eligible for inclusion in the adjusted model, and only variables with *p* ≤ 0.05 were retained in the final model. All statistical analyses were performed using Stata software (version 16.0; StataCorp, College Station, TX, USA). Due to the small number of included studies, regression analyses are presented only as exploratory findings in the Table S7.

## 3 Results

**FIGURE 1:**
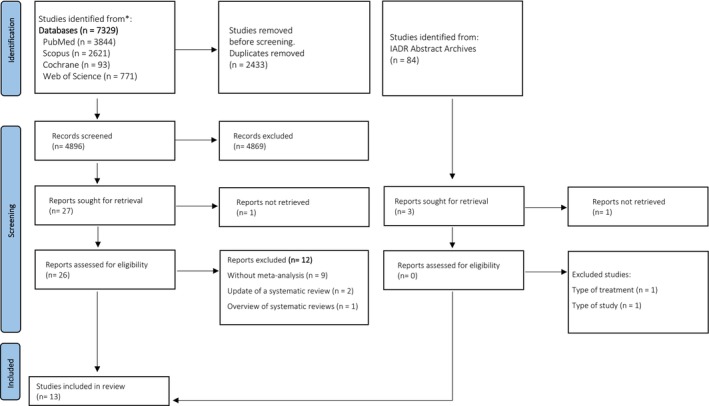
Flow diagram of study selection.

A total of 7329 articles were identified across all databases. After the removal of 2433 duplicates, 4896 records remained for title and abstract screening. Additionally, a manual search of the grey literature (IADR Abstract Archives) yielded 84 potentially relevant studies; however, none met the inclusion criteria. Following screening, 27 studies were selected for full‐text review, but one could not be retrieved. Of the remaining 26 systematic reviews, 13 met the inclusion criteria and were included in the final analysis [1, 2, 31, 32, 33, 34, 35, 36, 37, 38, 39, 40, 41](Figure 1). When the full text was not available through institutional subscriptions or alternative sources, two attempts were made to contact the corresponding author via email. Despite these efforts, no response was received, and one article could not be retrieved; therefore, it was excluded from the final analysis.

### 3.1 Characteristics of Included Studies

Table 1 summarises the main characteristics of the included studies. The year of publication ranged from 2005 to 2024, with the majority published before 2021 (*n* = 7). Most studies originated from Asia (*n* = 6). Eleven of the 13 studies adhered to the journal guidelines regarding abstract word limits and correctly identified both primary and secondary outcomes. Five studies did not report any funding source, and five disclosed conflicts of interest. Seven studies cited the PRISMA statement as the reporting guideline, ten reported the use of a tool to assess risk of bias, four evaluated the quality of evidence using the GRADE approach, and seven registered a protocol for their systematic review.

**TABLE 1:** Characteristics of the included studies (*n* = 13).

Characteristics	Total (%)	Spin in full text mean (SD)	*p*	Spin in abstract mean (SD)	*p*
Year					
≤ 2020	6 (46.15%)	3.83 (2.13)	0.018^a^	3.50 (1.97)	0.332^a^
≥ 2021	7 (53.85%)	1.14 (1.34)		2.28 (2.80)	
Continent					
Asia	6 (46.15%)	2.50 (2.07)	0.197^b^	3.00 (2.10)	0.583^b^
Europe	3 (23.08%)	0.66 (0.57)		1.33 (1.15)	
Oceania	1 (7.69%)	6.00 (0.00)		4.00 (0.00)	
America	3 (23.08%)	2.66 (2.51)		3.66 (3.05)	
Compliance with word limit					
No	2 (15.38%)	5.50 (0.70)	0.603^a^	4.00 (2.64)	0.232^a^
Yes	11 (84.62%)	1.81 (1.83)		2.50 (2.01)	
Primary/secondary outcome defined					
No	2 (15.38%)	5.50 (0.70)	0.707^a^	5.50 (2.12)	0.280^a^
Yes	11 (84.62%)	1.81 (1.83)		2.36 (1.85)	
Funding source					
None	6 (46.15%)	3.83 (2.31)	0.289^b^	4.33 (2.25)	0.225^b^
Nonprofit	2 (15.38%)	0.50 (0.70)		1.00 (1.41)	
Not reported	5 (38.46%)	1.40 (1.14)		1.80 (0.83)	
PRISMA cited					
No	6 (46.15%)	2.83 (2.31)	0.427^a^	2.83 (2.40)	0.378^a^
Yes	7 (53.85%)	2.00 (2.16)		2.85 (2.11)	
Open access					
No	3 (23.08%)	2.83 (2.31)	0.427^a^	2.83 (2.40)	0.378^a^
Yes	10 (76.92%)	2.00 (2.16)		2.85 (2.11)	
Outcome result					
Negative	10 (76.92%)	2.90 (2.23)	0.064^b^	3.30 (2.26)	0.062^b^
Positive	3 (23.08%)	0.66 (0.57)		1.33 (0.57)	
Structured abstract					
No	3 (23.08%)	2.33 (3.21)	0.132^b^	2.66 (1.15)	0.804^b^
Yes	10 (76.92%)	2.40 (2.01)		2.90 (2.42)	
Conflict of interest					
No	8 (61.54%)	2.50 (2.44)	0.335^a^	2.50 (1.06)	0.995^a^
Yes	5 (38.46%)	2.50 (1.92)		3.40 (3.36)	
Risk of bias assessment					
No	3 (23.08%)	5.66 (0.57)	0.815^a^	5.00 (0.69)	0.457^a^
Yes	10 (76.92%)	1.40 (1.26)		2.20 (1.87)	
GRADE used					
No	9 (69.23%)	3.00 (2.23)	0.243^a^	3.44 (1.72)	0.196^a^
Yes	4 (30.77%)	1.00 (1.41)		1.50 (1.29)	
Protocol registered					
No	6 (46.15%)	3.83 (2.13)	0.145^a^	3.33 (1.68)	0.515^a^
Yes	7 (53.85%)	1.14 (1.34)		2.42 (2.16)	

^a^ *T* student.

^b^ One‐way test.

### 3.2 Occurrence of Spin

In the full‐text analysis, the most prevalent spin strategy was failure to acknowledge departures from the review protocol that could influence the interpretation of results (*n* = 6). In abstracts, the most common spin strategy was failure to report the number of studies or participants contributing to the main outcomes (*n* = 9), followed by conclusions claiming equivalence based on non‐statistically significant findings with wide confidence intervals (*n* = 4).

A high prevalence of spin was observed in both abstracts (*n* = 12) and full texts (*n* = 10). The total number of spin identified per systematic review ranged from 0 to 6 in the full text and from 0 to 7 in the abstract. More than one type of spin was present in seven studies of full texts and 10 studies of the abstracts assessment. Among the 13 included studies, only three did not present any type of spin in the full text; in abstracts, only one review. Concerning the level of spin in abstracts, which considers the presence of more severe forms of spin, three reviews (*n* = 3) were classified as having a high level of spin, nine reviews as having a low level, and one review as having no spin (Table 1).

### 3.3 Factors Associated With the Occurrence of Spin

The adjusted Poisson regression analysis identified factors associated with a higher number of spin in the main text of systematic reviews (Table S7). Negative outcome results were associated with a 3.75 times higher likelihood of spin (95% CI: 1.55–9.05). Finally, the absence of a bias risk assessment was linked to a 2.83 times higher risk of spin (95% CI: 1.22–6.55).

### 3.4 Assessment of the Methodological Quality of the Systematic Reviews

**FIGURE 2:**
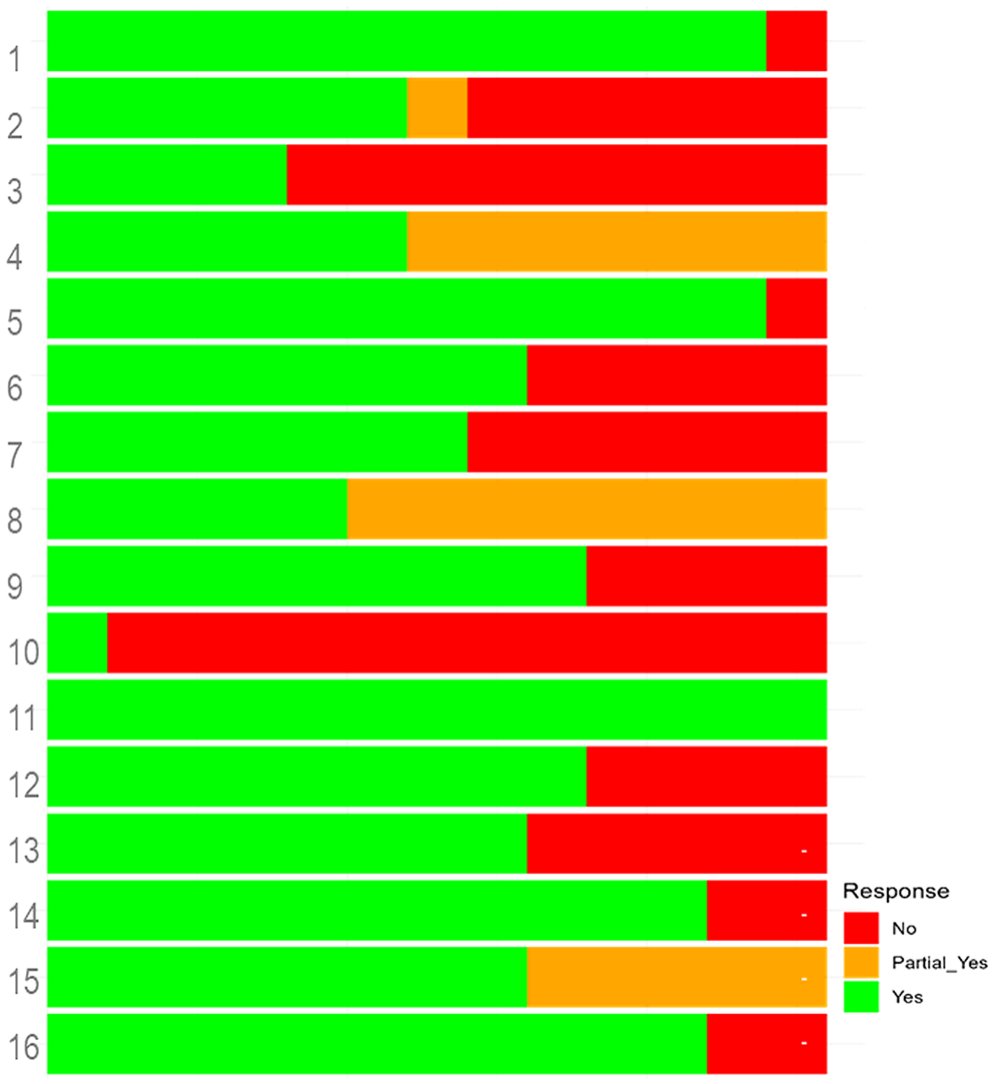
Quality assessment of the included systematic reviews—AMSTAR 2. 1. Components of PICO? 2. Review protocol? 3. Explanation for study design? 4. Comprehensive literature search strategy? 5. Study selection in duplicate? 6. Data extraction in duplicate? 7. List of excluded studies and justify the exclusions? 8. Describe the included studies in adequate detail? 9. Satisfactory technique for assessing the risk of bias? 10. Report on the sources of funding? 11. Appropriate methods for statistical combination? 12. Assess the potential impact of RoB? 13. Account for RoB when interpreting? 14. Explanation for and discussion of any heterogeneity? 15. Investigation of publication bias? 16. Report any potential sources of conflict of interest?

Figure 2 summarises the methodological quality of the included systematic reviews, evaluated with the AMSTAR 2 instrument. All reviews employed appropriate statistical methods for data synthesis. Most clearly specified their PICO components, conducted study selection in duplicate, examined heterogeneity among the included studies, and disclosed conflicts of interest. Only one review fully reported the funding source for each primary study and was therefore the sole review to satisfy every AMSTAR 2 criterion. Conversely, one review failed to meet all the criteria. Based on the AMSTAR 2 overall confidence ratings, eight reviews were judged to be of critically low quality, two of low quality, one of moderate quality, and two of high quality. Table 2 presents the individual characteristics of the included systematic reviews, including the authors' conclusions alongside assessments of quality.

**TABLE 2:** Individual characteristics of the included systematic reviews.

Author and year	Outcome	Conclusion	Notes	Level of spin in abstract	Number of spin	Spin in main text
Almeida et al., 2017	Postoperative pain Periapical healing and microbiological control	Multiple‐session treatment resulted in higher postoperative pain. No statistical difference between groups in periapical healing and microbiological control.	*N* = 17. Did not assess risk of bias or quality of evidence. AMSTAR: Very low‐quality review.	HIGH	7	5
Izadpanah et al., 2021	Postoperative pain	Single‐session treatment resulted in higher postoperative pain.	*N* = 27. Acceptable statistical heterogeneity. Risk of bias: no publication bias. Moderate evidence. AMSTAR: Very low‐quality review.	LOW	1	1
Mergoni et al., 2022	Extraction Radiographic failure Post‐treatment pain. Immediate post‐obturation pain Late post‐obturation pain Edema and flare‐up Use of analgesics Presence of fistula	1. No evidence that one technique is better than the other. 2. Uncertain evidence about extractions for endodontic reasons. 3. No difference in radiographic outcomes up to 1 year. Pain in the first week was higher in the single‐session group. 4. No apparent difference in pain incidence. 5–8. Uncertain evidence on the effects of treatment on these outcomes.	*N* = 47. Most studies had unclear or high risk of bias. Very low‐quality evidence. Moderate‐quality evidence. Moderate‐quality evidence. Low‐quality evidence. Very low‐quality evidence6. Very low‐quality evidence7. Very low‐quality evidence8. Very low‐quality evidence AMSTAR: High‐quality review	NONE	0	0
Nascimento et al., 2021	Reduction or elimination of endotoxins	Multiple‐session treatment was more effective in reducing endotoxins with medication acting for 14 or 30 days. No difference between groups for single‐session vs. 7‐day medication.	*N* = 9. Low risk of bias. Did not assess quality of evidence. AMSTAR: High‐quality review.	LOW	2	1
Nunes et al., 2021	Postoperative pain in retreatments	No difference between groups, except for mild pain, which was higher in the multiple‐session group.	*N* = 4. Low risk of bias. Low‐quality evidence. AMSTAR: Low‐quality review.	LOW	3	3
Sathorn et al., 2005	Clinical and radiographic success (healing) rate	Single‐session treatment seemed more effective for healing rate than multiple‐session, but not statistically significant.	*N* = 3. Did not assess risk of bias or quality of evidence. AMSTAR: Very low‐quality review.	HIGH	4	6
Scardini et al., 2023	Postoperative pain in retreatments	No difference in postoperative pain between retreatment techniques, number of visits or use of solvent.	*N* = 11; Meta‐analysis = 10. Low risk of bias. Very low‐quality evidence. AMSTAR: Low‐quality review.	LOW	1	0
Schwendicke and Göstemeyer, 2017	Risk of long‐term complications, postoperative pain and flare‐up	No difference between groups for risk of complications and postoperative pain. Significantly higher risk of flare‐up in single‐session treatment.	*N* = 29. High risk of bias. Complications: low‐quality evidence. Pain: moderate‐quality evidence. Flare‐up: very low‐quality evidence. AMSTAR: Moderate‐quality review.	LOW	2	1
Su et al., 2011	Healing rate and postoperative pain	No difference in healing rate and medium‐term postoperative pain. Multiple‐session treatment resulted in higher short‐term postoperative pain.	*N* = 10; Risk of bias: low (4), moderate (3), high (3). Did not assess quality of evidence. AMSTAR: Very low‐quality review.	LOW	2	2
Swapna et al., 2024	Postoperative pain and adverse effects in retreatments	Multiple‐session treatment resulted in higher postoperative pain.	*N* = 3; Risk of bias: unclear (2), high (1). Did not assess quality of evidence. AMSTAR: Very low‐quality review.	HIGH	7	3
Vishwanathaiah et al., 2021	Postoperative pain and risk of flare‐up	No difference between groups in postoperative pain and flare‐up risk.	*N* = 21; Moderate risk of bias. Did not assess quality of evidence. AMSTAR: Very low‐quality review.	LOW	2	0
Wong et al., 2014	Postoperative complications and healing (success) rate	No difference between groups in postoperative complications and healing (success) rate.	*N* = 47; Meta‐analysis = 27. Did not assess risk of bias or quality of evidence. AMSTAR: Very low‐quality review.	LOW	4	6
Zhang et al., 2015	Healing (success) rate	No difference between groups in healing (success) rate.	*N* = 9; Unclear risk of bias. Did not assess quality of evidence. AMSTAR: Very low‐quality review.	LOW	2	3

*Note: N* = number of primary studies included in each systematic review. AMSTAR rating refers to overall methodological quality according to AMSTAR 2. Level of spin in abstract was classified as none, low or high based on the presence and severity of predefined spin strategies. Number of spin indicates the total count of spin strategies identified in the abstract. Spin in main text indicates the total number of spin strategies identified in the full text.

## 4 Discussion

The assessment of spin in scientific literature is a relatively recent practice, first introduced in 2010 [10]. Since then, numerous studies have critically examined the presence of spin, focusing not only on its frequency but also on its potential influence within the biomedical literature. By 2016, although a specific classification tool had been developed to identify spin in systematic reviews and meta‐analyses, no published reports had yet documented its occurrence in these types of studies. The first evaluation of spin in systematic reviews and meta‐analyses in dentistry appears to have been published in 2021 in the field of orthodontics [25], followed by an investigation in endodontics in 2022 [24]. Before these, several publications had already highlighted the presence and consequences of spin in randomised clinical trials [42, 43]. Given the established evidence of spin in randomised controlled trials, it is reasonable to assume that similar distortions may influence the synthesis of evidence in systematic reviews, with or without meta‐analysis.

A high prevalence of spin was identified in both abstracts (*n* = 12) and full texts (*n* = 10). It is important to note, however, that spin was classified as low‐level in 69% of the abstracts, indicating that none of the nine most severe types of spin were detected. Interestingly, the total number of spin occurrences showed greater variation in abstracts, despite the smaller number of criteria assessed in this section. This may be related to the role of the abstract as a promotional component of the article, intended to attract readers' attention by providing a concise and engaging overview of the study, thereby encouraging further reading of the full text [42].

In this context, researchers may be more likely to introduce spin into manuscripts due to the highly competitive nature of contemporary science, which often fosters detrimental practices within the ‘publish or perish’ culture driven by productivity metrics and academic impact. Furthermore, evidence suggests that not only researchers but also peer reviewers and journal editorial boards tend to favour the publication of statistically significant results, which may contribute to the distortion or overinterpretation of findings [21].

In endodontics, only a few studies to date have investigated the presence of spin. Similar to research in other areas of dentistry, these studies have primarily focused on the analysis of abstracts and randomised controlled trials [24, 42, 43, 44]. The evaluation of spin in abstracts is particularly relevant, as many readers, both researchers and clinicians, have limited access to full‐text articles and often rely solely on abstracts to inform their understanding and, in some cases, clinical decisions. Giannakoulas et al. [24] assessed the prevalence of spin in systematic reviews and meta‐analyses in endodontics, restricting their evaluation to abstracts only. Spin was identified in over 67% of the abstracts, with most exhibiting more than one type of spin, findings that align closely with those of the present study.

To the best of our knowledge, this is the first study in endodontics to assess spin not only in abstracts but also in the full text, and only the second to examine spin in systematic reviews with meta‐analyses within the field. Such a comprehensive evaluation is essential, as increased awareness of these biases is expected to drive improvements in transparency and clarity, ultimately contributing to higher standards in both research and clinical practice. Moreover, our findings indicate that the practice of spin is not confined to abstracts alone, highlighting the necessity of evaluating full texts as well.

The identification of spin is often not straightforward, representing an inherent limitation of this type of study. Although standardised instruments for data collection, evaluation and classification of spin are available, interpretation still involves a certain degree of subjectivity [11]. To ensure consistency, all assessments were performed in duplicate, and any disagreements were resolved by consensus, with a third reviewer contributing when necessary [44]. Moreover, spin can manifest in various forms, each with different levels of severity and potential implications [10]. Accordingly, this analysis employed duplicate data extraction, and in cases of discordance, a third reviewer contributed to the final classification to minimise subjectivity in data collection and interpretation. Nevertheless, even with independent assessments, individual perspectives and pre‐existing beliefs may influence how spin is interpreted. Additionally, the relatively small number of studies included in the analysis represents another limitation, as it may increase the risk of a type II error, that is, failing to detect an existing association. To mitigate this, statistical methods more suitable for small samples, such as Firth's penalised logistic regression, were applied to obtain more stable and unbiased estimates for an exploratory analysis [26, 30].

The prevalence of spin reported in the literature is moderate to high [12, 44, 45]. One factor that appears to be consistently associated with the presence of spin is the reporting of non‐statistically significant results [45]. This pattern was also observed in the present study, where spin frequently occurred through the misinterpretation or misrepresentation of non‐significant outcomes, particularly when such results were described as equivalent between groups. These instances were especially common in the conclusion sections of systematic reviews, reflecting a recurrent tendency to frame findings more favourably than the data actually support.

The occurrence of spin can vary in both frequency and severity, each with potentially different implications. It is often unclear whether spin arises from unintentional error, limited methodological skill or deliberate intent. Given the uncertain impact of spin on research and clinical practice, and the unclear factors contributing to it, reporting prevalence alone may be insufficient [11, 46]. Additionally, because study quality can influence outcomes and lead to differing conclusions, this study also assessed the methodological quality of the included systematic reviews [23].

It is important to consider that reporting standards evolve over time. Older systematic reviews, such as those published before recent updates in reporting guidelines, should be interpreted within the methodological context of their publication period. In this study, year of publication was explored to assess potential temporal patterns in spin prevalence, including a comparison before and after the PRISMA 2020 update [15]. However, this analysis was exploratory and not intended to judge earlier publications by standards that were not yet established.

Misleading conclusions can create the false impression that treatments are equivalent or comparable, even when the study was not designed to test equivalence or non‐inferiority. In this study, most of the included systematic reviews were rated as low or critically low quality according to the AMSTAR 2 tool, suggesting that methodological weaknesses and potential bias may have compromised the reliability of their findings [28]. As a result, the current evidence does not clearly indicate whether single‐ or multiple‐visit root treatments are superior, inferior or equivalent. Among the 13 reviews analysed, eight reported statistically significant differences between treatment types, four favouring multiple visits and four favouring single visits, while five found no statistically significant differences. Reviews with lower levels of spin and higher methodological quality generally concluded that the available evidence remains insufficient to determine which approach performs better.

Although the qualitative assessment allowed for a detailed examination of spin patterns within a focused topic in endodontics, the small number of included reviews limits the statistical power of exploratory association analyses. Any observed statistical associations should therefore be interpreted cautiously, as the limited sample size increases the risk of imprecision and model instability. The regression findings are exploratory and hypothesis‐generating rather than confirmatory. Overall, these findings contribute to the ongoing discussion on spin and underscore the need for more transparent and methodologically robust evidence synthesis in this field.

It is important to emphasise that spin is not always the sole explanation for the discrepancies observed among systematic reviews. Differences in inclusion criteria, methodological rigour or analytical approaches may also lead to divergent conclusions regarding the superiority of single‐versus multiple‐visit endodontic treatments [11]. In some cases, inconsistencies among reviews may reflect the limited or heterogeneous nature of the available evidence rather than intentional or unintentional distortion of results. Therefore, both methodological quality and the presence of spin should be critically evaluated on an individual basis before any direct comparison between reviews is made. A low‐quality review that also presents severe spin is already an indication that its conclusions may be unreliable or misleading [10]. Furthermore, the existing evidence base may still be insufficient to establish definitive clinical recommendations on whether single‐ or multiple‐visit root canal treatments are superior, reinforcing the need for well‐designed randomised controlled trials and transparent systematic reviews [1].

## 5 Conclusion

The occurrence of spin in both abstracts and full texts was high, although most reviews presented low‐level spin in abstracts. Overall, the methodological quality of the included systematic reviews was predominantly low or critically low according to AMSTAR 2. Notably, reviews with lower levels of spin and higher methodological quality generally concluded that the available evidence remains insufficient to determine the superiority of single‐ or multiple‐visit root canal treatments. In contrast, more definitive claims were more frequently observed in reviews with lower methodological rigour or greater presence of spin. These findings highlight the importance of critically appraising both methodological quality and reporting practices when interpreting evidence in endodontics and underscore the need for more transparent and robust evidence synthesis.

## Author Contributions

**A.L.C.P.:** conceptualisation, data collection, analysis, drafting the manuscript, methodological assessment, writing – original draft. **L.G.‐S.:** conceptualisation, data collection, analysis, data extraction, methodological assessment, writing – original draft. **B.L.:** study design, statistical analysis, writing – original draft. **F.G.P.:** conceptualisation, study design, statistical analysis, supervised the project, writing – original draft. **L.A.C.:** study design, statistical analysis, writing – original draft. FGP: conceptualisation, supervised the project, writing – original draft. **T.P.‐C.:** conceptualisation, data collection, supervised the project, writing – original draft. All Authors approved the final manuscript and agree to be accountable for all aspects of the work.

## Funding

This work was partially financed by Coordenação de Aperfeiçoamento de Pessoal de Nível Superior (CAPES), Finance Code 001.

## Disclosure

Protocol registration: https://osf.io/wxnp8/.

## Conflicts of Interest

The authors declare no conflicts of interest.

## Supporting information


**Supporting Information: S1** PRISMA 2020 checklist.

**Supporting Information: S2** Electronic databases and search strategy.

**Supporting Information: S3** Reports excluded and reasons.

**Supporting Information: S4** Variables extracted and analysed in the meta‐research study.

**Supporting Information: S5** Categorisation of spin in the main body and abstract sections of systematic reviews and meta‐analyses (adapted from Yavchitz et al. 2016).

**Supporting Information: S6** Type of spin in abstracts of systematic reviews from most to least severe (based on Yavchitz et al. 2016).

**Supporting Information: S7** Poisson regression analysis of potential predictors of the number of spin occurrences in the full text of systematic reviews (robust RR).

## Data Availability

The data that supports the findings of this study are available in the Supporting Information of this article.
