# Supplementary material for: Spin and Methodological Quality of Systematic Reviews and Meta‐Analyses Comparing Single‐ and Multiple‐Visit Endodontic Treatment: A Meta‐Research Study
Source: Aust Endod J. 2026 May 22;52(2):654–65. doi: 10.1111/aej.70087 (PMC13436167; doi:10.1111/aej.70087)
Supplement: Supplementary file 1 [file AEJ-52-654-s001.docx]

**Supplementary Table 7.** Poisson regression analysis of potential predictors of the number of spin occurrences in the full text of systematic reviews (robust RR).

|  | **Spin in Main Text** | | | |
| --- | --- | --- | --- | --- |
| **Variable** | **RR (Unadjusted)** | **p-value** | **RR (Adjusted)** | **p-value** |
| **Year** |  |  |  |  |
| ≥ 2021 | 1 | 0.011 |  |  |
| ≤ 2020 | 3.35 (1.30 – 8.59) |  |  |  |
| **Word Count Compliance** | |  |  |  |
| Yes | 1 | 0.666 |  |  |
| No | 1.25 (0.45 – 3.44) |  |  |  |
| **Primary/Secondary Outcome Specified** | | |  |  |
| Yes | 1 | <0.001 |  |  |
| No | 3.02 (1.65 – 5.54) |  |  |  |
| **Funding Source** | | |  |  |
| Non-profit | 1 | 0.004 | 1 | 0.094 |
| None | 7.66 (1.68 – 34.84) |  | 4.00 (0.75 – 21.15) |  |
| Not reported | 2.80 (0.57 – 13.70) |  | 5.00 (1.14 – 21.76) |  |
| **PRISMA Citation** | |  |  |  |
| Yes | 1 | 0.490 |  |  |
| No | 1.41 (0.52 – 3.81) |  |  |  |
| **Open Access** |  |  |  |  |
| Yes | 1 | 0.165 |  |  |
| No | 1.83 (0.70 – 4.32) |  |  |  |
| **Outcome Result** |  |  |  |  |
| Positive | 1 | 0.002 | 1 | 0.003 |
| Negative | 4.35 (1.67 – 11.32) |  | 3.75 (1.55 – 9.05) |  |
| **Structured Abstract** | |  |  |  |
| Yes | 1 | 0.969 |  |  |
| No | 0.97 (0.23 – 4.02) |  |  |  |
| **Conflict of Interest** | |  |  |  |
| Yes | 1 | 0.796 |  |  |
| No | 1.13 (0.42 – 3.00) |  |  |  |
| **Risk of Bias** |  |  |  |  |
| Yes | 1 | <0.001 | 1 | 0.015 |
| No | 4.04 (2.30 – 7.09) |  | 2.83 (1.22 – 6.55) |  |
| **GRADE Usage** |  |  |  |  |
| Yes | 1 | 0.107 |  |  |
| No | 3.00 (0.78 – 11.42) |  |  |  |
| **Protocol Registration** | |  |  |  |
| Yes | 1 | 0.117 |  |  |
| No | 3.35 (1.30 – 8.59) |  |  |  |
