# Supplementary material for: Spin and Methodological Quality of Systematic Reviews and Meta‐Analyses Comparing Single‐ and Multiple‐Visit Endodontic Treatment: A Meta‐Research Study
Source: Aust Endod J. 2026 May 22;52(2):654–65. doi: 10.1111/aej.70087 (PMC13436167; doi:10.1111/aej.70087)
Supplement: Supplementary file 2 [file AEJ-52-654-s002.docx]

**Supplementary Material 2.** Electronic Databases and Search Strategy.

| **Electronic Database** | **Search Strategy** |
| --- | --- |
| **PubMed MEDLINE**  **3,844** | ((Endodontics) OR (Endodontology) OR (Root Canal Treatment) OR (Canal Therapies, Root) OR (Canal Therapy, Root) OR (Root Canal Therapies) OR (Therapies, Root Canal) OR (Therapy, Root Canal) OR (Root Canal Preparation) OR (Preparation, Root Canal) OR (Root Canal Preparations) OR (Access Cavity Preparation) OR (Access Cavity Preparations) OR (Preparation, Access Cavity) OR (Endodontics Access Cavity Preparation) OR (Pulpectomy) OR (Pulpectomies) OR (Dental Pulp Devitalization) OR (Devitalization, Dental Pulp) OR (Pulp Devitalization, Dental) OR (Root Canal Obturation) OR (Canal Obturation, Root) OR (Canal Obturations, Root) OR (Obturation, Root Canal) OR (Obturations, Root Canal) OR (Root Canal Obturations) OR (Endodontic Obturation) OR (Endodontic Obturations) OR (Obturation, Endodontic) OR (Obturations, Endodontic) OR (Single-visit) OR (Single Visit) OR (Multiple-visit) OR (Multiple Visits) OR (One-appointment) OR (Conventional Multivisit Treatment)) AND ((Meta-Analysis) OR (Meta-Analysis as Topic) OR (Meta Analysis as Topic) OR (Data Pooling) OR (Data Poolings) OR (Overviews, Clinical Trial) OR (Clinical Trial Overviews) OR (Clinical Trial Overview) OR (Overview, Clinical Trial) OR (Network Meta-Analysis) OR (Meta-Analyses, Network) OR (Meta-Analysis, Network) OR (Network Meta Analysis) OR (Network Meta-Analyses) OR (Multiple Treatment Comparison Meta-Analysis) OR (Multiple Treatment Comparison Meta Analysis) OR (Mixed Treatment Meta-Analysis) OR (Meta-Analyses, Mixed Treatment) OR (Meta-Analysis, Mixed Treatment) OR (Mixed Treatment Meta Analysis) OR (Mixed Treatment Meta-Analyses) OR (meta analysis) OR (analysis, meta) OR (metaanalysis) OR (metaanalyses) OR (Systematic Reviews as Topic) OR (Systematic Review as Topic) OR (Reviews Systematic as Topic) OR (Systematic Review) OR (Review, Systematic) OR (systematic literature review) OR (systematic scoping review) OR (systematic narrative review) OR (systematic qualitative review) OR (systematic evidence review) OR (systematic quantitative review) OR (systematic meta-review) OR (systematic critical review) OR (systematic mixed studies review) OR (systematic mapping review) OR (systematic cochrane review) OR (systematic integrative review) OR (systematic reviews) OR (((systematic review[ti] OR systematic literature review[ti] OR systematic scoping review[ti] OR systematic narrative review[ti] OR systematic qualitative review[ti] OR systematic evidence review[ti] OR systematic quantitative review[ti] OR systematic meta-review[ti] OR systematic critical review[ti] OR systematic mixed studies review[ti] OR systematic mapping review[ti] OR systematic cochrane review[ti] OR systematic search and review[ti] OR systematic integrative review[ti]) NOT comment[pt] NOT (protocol[ti] OR protocols[ti])) NOT MEDLINE [subset]) OR (Cochrane Database Syst Rev[ta] AND review[pt]) OR systematic review[pt]) |
| **Cochrane Collaboration Library**  **93** | (Endodontics) OR (Endodontology) OR (Root Canal Treatment) OR (Canal Therapies, Root) OR (Canal Therapy, Root) OR (Root Canal Therapies) OR (Therapies, Root Canal) OR (Therapy, Root Canal) OR (Root Canal Preparation) OR (Preparation, Root Canal) OR (Root Canal Preparations) OR (Access Cavity Preparation) OR (Access Cavity Preparations) OR (Preparation, Access Cavity) OR (Endodontics Access Cavity Preparation) OR (Pulpectomy) OR (Pulpectomies) OR (Dental Pulp Devitalization) OR (Devitalization, Dental Pulp) OR (Pulp Devitalization, Dental) OR (Root Canal Obturation) OR (Canal Obturation, Root) OR (Canal Obturations, Root) OR (Obturation, Root Canal) OR (Obturations, Root Canal) OR (Root Canal Obturations) OR (Endodontic Obturation) OR (Endodontic Obturations) OR (Obturation, Endodontic) OR (Obturations, Endodontic) OR (Single-visit) OR (Single Visit) OR (Multiple-visit) OR (Multiple Visits) OR (One-appointment) OR (Conventional Multivisit Treatment) in Title Abstract Keyword AND (Meta-Analysis) OR (Meta-Analysis as Topic) OR (Meta Analysis as Topic) OR (Data Pooling) OR (Data Poolings) OR (Overviews, Clinical Trial) OR (Clinical Trial Overviews) OR (Clinical Trial Overview) OR (Overview, Clinical Trial) OR (Network Meta-Analysis) OR (Meta-Analyses, Network) OR (Meta-Analysis, Network) OR (Network Meta Analysis) OR (Network Meta-Analyses) OR (Multiple Treatment Comparison Meta-Analysis) OR (Multiple Treatment Comparison Meta Analysis) OR (Mixed Treatment Meta-Analysis) OR (Meta-Analyses, Mixed Treatment) OR (Meta-Analysis, Mixed Treatment) OR (Mixed Treatment Meta Analysis) OR (Mixed Treatment Meta-Analyses) OR (meta analysis) OR (analysis, meta) OR (metaanalysis) OR (metaanalyses) OR (Systematic Reviews as Topic) OR (Systematic Review as Topic) OR (Reviews Systematic as Topic) OR (Systematic Review) OR (Review, Systematic) OR (systematic literature review) OR (systematic scoping review) OR (systematic narrative review) OR (systematic qualitative review) OR (systematic evidence review) OR (systematic quantitative review) OR (systematic meta-review) OR (systematic critical review) OR (systematic mixed studies review) OR (systematic mapping review) OR (systematic cochrane review) OR (systematic integrative review) OR (systematic search and review) in Title Abstract Keyword |
| **Scopus**  **2,621** | ( endodontics ) OR ( endodontology ) OR ( root AND canal AND treatment ) OR ( canal AND therapies, AND root ) OR ( canal AND therapy, AND root ) OR ( root AND canal AND therapies ) OR ( therapies, AND root AND canal ) OR ( therapy, AND root AND canal ) OR ( root AND canal AND preparation ) OR ( preparation, AND root AND canal ) OR ( root AND canal AND preparations ) OR ( access AND cavity AND preparation ) OR ( access AND cavity AND preparations ) OR ( preparation, AND access AND cavity ) OR ( endodontics AND access AND cavity AND preparation ) OR ( pulpectomy ) OR ( pulpectomies ) OR ( dental AND pulp AND devitalization ) OR ( devitalization, AND dental AND pulp ) OR ( pulp AND devitalization, AND dental ) OR ( root AND canal AND obturation ) OR ( canal AND obturation, AND root ) OR ( canal AND obturations, AND root ) OR ( obturation, AND root AND canal ) OR ( obturations, AND root AND canal ) OR ( root AND canal AND obturations ) OR ( endodontic AND obturation ) OR ( endodontic AND obturations ) OR ( obturation, AND endodontic ) OR ( obturations, AND endodontic ) OR ( single-visit ) OR ( single AND visit ) OR ( multiple-visit ) OR ( multiple AND visits ) OR ( one-appointment ) OR ( conventional AND multivisit AND treatment ) AND ( meta-analysis ) OR ( meta-analysis AND as AND topic ) OR ( meta AND analysis AND as AND topic ) OR ( data AND pooling ) OR ( data AND poolings ) OR ( overviews, AND clinical AND trial ) OR ( clinical AND trial AND overviews ) OR ( clinical AND trial AND overview ) OR ( overview, AND clinical AND trial ) OR ( network AND meta-analysis ) OR ( meta-analyses, AND network ) OR ( meta-analysis, AND network ) OR ( network AND meta AND analysis ) OR ( network AND meta-analyses ) OR ( multiple AND treatment AND comparison AND meta-analysis ) OR ( multiple AND treatment AND comparison AND meta AND analysis ) OR ( mixed AND treatment AND meta-analysis ) OR ( meta-analyses, AND mixed AND treatment ) OR ( meta-analysis, AND mixed AND treatment ) OR ( mixed AND treatment AND meta AND analysis ) OR ( mixed AND treatment AND meta-analyses ) OR ( meta AND analysis ) OR ( analysis, AND meta ) OR ( metaanalysis ) OR ( metaanalyses ) OR ( systematic AND reviews AND as AND topic ) OR ( systematic AND review AND as AND topic ) OR ( reviews AND systematic AND as AND topic ) OR ( systematic AND review ) OR ( review, AND systematic ) OR ( systematic AND literature AND review ) OR ( systematic AND scoping AND review ) OR ( systematic AND narrative AND review ) OR ( systematic AND qualitative AND review ) OR ( systematic AND evidence AND review ) OR ( systematic AND quantitative AND review ) OR ( systematic AND meta-review ) OR ( systematic AND critical AND review ) OR ( systematic AND mixed AND studies AND review ) OR ( systematic AND mapping AND review ) OR ( systematic AND cochrane AND review ) OR ( systematic AND integrative AND review ) OR ( systematic AND search AND review ) |
| **Web of science**  **771** | (TS=("Endodontics" OR "Endodontology" OR "Root Canal Treatment" OR "Canal Therapies, Root" OR "Canal Therapy, Root" OR "Root Canal Therapies" OR "Therapies, Root Canal" OR "Therapy, Root Canal" OR "Root Canal Preparation" OR "Preparation, Root Canal" OR "Root Canal Preparations" OR "Access Cavity Preparation" OR "Access Cavity Preparations" OR "Preparation, Access Cavity" OR "Endodontics Access Cavity Preparation" OR "Pulpectomy" OR "Pulpectomies" OR "Dental Pulp Devitalization" OR "Devitalization, Dental Pulp" OR "Pulp Devitalization, Dental" OR "Root Canal Obturation" OR "Canal Obturation, Root" OR "Canal Obturations, Root" OR "Obturation, Root Canal" OR "Obturations, Root Canal" OR "Root Canal Obturations" OR "Endodontic Obturation" OR "Endodontic Obturations" OR "Obturation, Endodontic" OR "Obturations, Endodontic" OR "Single-visit" OR "Single Visit" OR "Multiple-visit" OR "Multiple Visits" OR "One-appointment" OR "Conventional Multivisit Treatment")) AND (TS=("Meta-Analysis" OR "Meta-Analysis as Topic" OR "Meta Analysis as Topic" OR "Data Pooling" OR "Data Poolings" OR "Overviews, Clinical Trial" OR "Clinical Trial Overviews" OR "Clinical Trial Overview" OR "Overview, Clinical Trial" OR "Network Meta-Analysis" OR "Meta-Analyses, Network" OR "Meta-Analysis, Network" OR "Network Meta Analysis" OR "Network Meta-Analyses" OR "Multiple Treatment Comparison Meta-Analysis" OR "Multiple Treatment Comparison Meta Analysis" OR "Mixed Treatment Meta-Analysis" OR "Meta-Analyses, Mixed Treatment" OR "Meta-Analysis, Mixed Treatment" OR "Mixed Treatment Meta Analysis" OR "Mixed Treatment Meta-Analyses" OR "meta analysis" OR "analysis, meta" OR "metaanalysis" OR "metaanalyses" OR "Systematic Reviews as Topic" OR "Systematic Review as Topic" OR "Reviews Systematic as Topic" OR "Systematic Review" OR "Review, Systematic" OR "systematic literature review" OR "systematic scoping review" OR "systematic narrative review" OR "systematic qualitative review" OR "systematic evidence review" OR "systematic quantitative review" OR "systematic meta-review" OR "systematic critical review" OR "systematic mixed studies review" OR "systematic mapping review" OR "systematic cochrane review" OR "systematic integrative review" OR "systematic search and review")) |
| **IADR Abstract Archives** | “single visit” 56 resultados  “multiple visit” 28 resultados |
