# Supplementary material for: Spin and Methodological Quality of Systematic Reviews and Meta‐Analyses Comparing Single‐ and Multiple‐Visit Endodontic Treatment: A Meta‐Research Study
Source: Aust Endod J. 2026 May 22;52(2):654–65. doi: 10.1111/aej.70087 (PMC13436167; doi:10.1111/aej.70087)
Supplement: Supplementary file 3 [file AEJ-52-654-s003.docx]

**Supplementary Material 5.** Categorization of Spin in the Main Body and Abstract Sections of Systematic Reviews and Meta-Analyses (Adapted from Yavchitz et al., 2016).

| **Principal Category** | **Exposition of spin in the main text** | **Exposition of spin in abstract** |
| --- | --- | --- |
| **MISLEADING REPORTING** | 1. Failure to acknowledge a departure from protocol that could modify the interpretation of results. 2. Selective reporting of or overemphasis on efficacy outcomes favoring the beneficial effect of the experimental intervention (e.g., secondary outcomes, subgroup analyses). 3. Selective reporting of or overemphasis on harm outcomes favoring the safety of the experimental intervention. 4. No or inadequate reporting of the limitations of the systematic review 5. Selective citation of articles in favor of the beneficial effect of the experimental intervention. 6. Authors hide or do not present any conflict of interest. 7. Conclusion focusing selectively on statistically significant efficacy outcome. 8. Selective reporting of analysis favoring the beneficial effect of the experimental intervention (e.g., selective analysis using random or fixed effect according the results). 9. Inadequate focus on the results of primary studies favoring the beneficial effect of the experimental intervention instead of the meta-analysis results. 10. Changing the scale of the forest plot to magnify the results (diamond size). | 1. Selective reporting of or overemphasis on efficacy outcomes favoring the beneficial effect of the experimental intervention. 2. Selective reporting of or overemphasis on harm outcomes favoring the safety of the experimental intervention. 3. Failure to report a wide confidence interval of estimates. 4. Authors hide or do not present any conflict of interest. 5. Inadequate focus on the results of primary studies favoring the beneficial effect of the experimental intervention instead of the meta-analysis results. 6. Conclusion focusing selectively on statistically significant efficacy outcome 7. Failure to report the number of studies/patients contributing to the analysis for main outcomes 8. Failure to specify the direction of the effect when it favors the control intervention. |
| **MISLEADING INTERPRETATION** | 11. Title claims or suggests a beneficial effect of the experimental intervention not supported by the findings. 12. Inadequate interpretation of nonstatistically significant results (with a wide confidence interval) as a lack of effect or an equivalent effect for efficacy outcomes. 13. Inadequate interpretation of nonstatistically significant results (with a wide confidence interval) as demonstrating safety for harm outcome. 14. Inadequate focus on P-value instead of magnitude of the effect estimates for harm or efficacy outcome. 15. Focus on relative effect when the absolute effect is small. 16. Misleading interpretation of cited articles, favoring the beneficial effect of the experimental intervention. 17. Conclusion claiming equivalence or comparable effectiveness for nonstatistically significant results with a wide confidence interval. 18. Conclusion formulating recommendations for clinical practice not supported by the findings. 19. Conclusion claiming safety based on nonstatistically significant results with a wide confidence interval. 20. Conclusion ignoring the high risk of bias of the studies, the heterogeneity or the reporting bias (i.e., alow level of evidence) in the interpretation of the results. 21. No or inadequate consideration of heterogeneity in results interpretation 22. No or inadequate consideration of the risk of bias of primary studies included in results interpretation (i.e., no reporting of the risk of bias of the primary studies, claiming the low risk of bias of studies included not supported by the data, no downgrading the evidence despite several high risk of bias studies). 23. No or inadequate consideration of reporting bias in results interpretation | 9. Title claims or suggests a beneficial effect of the experimental intervention not supported by the findings. 10. Inadequate focus on P-value instead of magnitude of the effect estimates for harm or efficacy outcome. 11. Focus on relative effect when the absolute effect is small. 12. Conclusion claiming equivalence or comparable effectiveness for non-statistically significant results with a wide confidence interval. 13. Conclusion formulating recommendations for clinical practice not supported by the findings. 14. Conclusion claiming safety based on nonstatistically significant results with a wide confidence interval. 15. Conclusion claiming the beneficial effect of the experimental treatment despite high risk of bias in primary studies. 16. Conclusion claiming the beneficial effect of the experimental treatment despite reporting bias. 17. Conclusion claiming the beneficial effect of the experimental treatment despite high heterogeneity. 18. Ignoring that the review included different study design (e.g., controlled trial or observational studies). |
| **INAPPROPRIATE EXTRAPOLATION** | 24. Inadequate extrapolation of the results from surrogate markers or specific outcome to the global improvement of the disease. 25. Inadequate extrapolation of the results to a larger population, a larger setting or a wider set of interventions (e.g., from a specific rehabilitation program to all rehabilitation programs, to a specialized unit to a nonspecialized medical unit etc). 26. Conclusion extrapolating the review’s findings to a different population or setting. 27. Conclusion extrapolating the review’s findings to a different intervention (i.e., claiming efficacy of one specific intervention although the review covers a class of several interventions). 28. Conclusion extrapolating the review’s findings from a surrogate marker or a specific outcome to the global improvement of the disease. | 19. Conclusion extrapolating the review’s findings to a different population or setting. 20. Conclusion extrapolating the review’s findings to a different intervention (i.e., claiming efficacy of one specific intervention although the review covers a class of several interventions). 21. Conclusion extrapolating the review’s findings from a surrogate marker or a specific outcome to the global improvement of the disease. |
