# Supplementary material for: Spin and Methodological Quality of Systematic Reviews and Meta‐Analyses Comparing Single‐ and Multiple‐Visit Endodontic Treatment: A Meta‐Research Study
Source: Aust Endod J. 2026 May 22;52(2):654–65. doi: 10.1111/aej.70087 (PMC13436167; doi:10.1111/aej.70087)
Supplement: Supplementary file 5 [file AEJ-52-654-s005.docx]

**Supplementary Table 6.** Type of spin in abstracts of systematic reviews from most to least severe (based on Yavchitz et al., 2016).

| Severity ranking | Description of spin |
| --- | --- |
| 1 | Conclusion contains recommendations for clinical practice not supported by the findings |
| 2 | Title claims or suggests a beneficial effect of the experimental intervention not supported by the findings |
| 3 | Selective reporting of or overemphasis on efficacy outcomes or analysis favoring the beneficial effect of the experimental intervention |
| 4 | Conclusion claims safety based on non-statistically significant results with a wide confidence interval |
| 5 | Conclusion claims the beneficial effect of the experimental treatment despite high risk of bias in primary studies |
| 6 | Selective reporting of or overemphasis on harm outcomes or analysis favoring the safety of the experimental intervention |
| 7 | Conclusion extrapolates the review’s findings to a different intervention (i.e., claiming efficacy of one specific intervention although the review covers a class of several interventions) |
| 8 | Conclusion extrapolates the review’s findings from a surrogate marker or a specific outcome to the global improvement of the disease |
| 9 | Conclusion claims the beneficial effect of the experimental treatment despite reporting bias |
