# Supplementary material for: Spin and Methodological Quality of Systematic Reviews and Meta‐Analyses Comparing Single‐ and Multiple‐Visit Endodontic Treatment: A Meta‐Research Study
Source: Aust Endod J. 2026 May 22;52(2):654–65. doi: 10.1111/aej.70087 (PMC13436167; doi:10.1111/aej.70087)
Supplement: Supplementary file 6 [file AEJ-52-654-s006.docx]

**Supplementary Material 3.** Reports excluded and reasons

| **Author and Year** | **Reason for Exclusion** |
| --- | --- |
| 1. Baeza Palacios et al., 2020 | No meta-analysis |
| 2. Bryce et al., 2023 | Commentary on Mergoni et al., 2022 |
| 3. Chaudhari et al., 2023 | No meta-analysis |
| 4. Choudhari et al., 2024 | No meta-analysis |
| 5. de-Deus et al., 2017 | No meta-analysis |
| 6. Drouri et al., 2024 | No meta-analysis |
| 7. Figini et al., 2007 | Update: Mergoni et al., 2022 |
| 8. Khabadze et al., 2021 | No meta-analysis |
| 9. Kumar et al., 2025 | No meta-analysis |
| 10. Manfredi et al., 2016 | Update: Mergoni et al., 2022 |
| 11. Moreira et al., 2017 | Overview of systematic reviews |
| 12. Rosso et al., 2012 | No meta-analysis |
| 13. Sathorn et al., 2007 | No meta-analysis |
| 14. Wang et al., 2017 | Not found |
