# Supplementary material for: Spin and Methodological Quality of Systematic Reviews and Meta‐Analyses Comparing Single‐ and Multiple‐Visit Endodontic Treatment: A Meta‐Research Study
Source: Aust Endod J. 2026 May 22;52(2):654–65. doi: 10.1111/aej.70087 (PMC13436167; doi:10.1111/aej.70087)
Supplement: Supplementary file 7 [file AEJ-52-654-s007.docx]

**Supplementary Table 4.** Variables extracted and analyzed in the meta-research study

| Category | Variable Name | Description / Measurement |
| --- | --- | --- |
| Bibliometric characteristics | Author | First author’s last name and initials |
|  | Year | Year of publication of the review |
|  | Title | Full title of the systematic review |
|  | Country (First Author) | Country of affiliation of the first author |
|  | Continent (First Author) | Continent where the first author is based |
|  | Journal | Journal in which the review was published |
|  | CiteScore (Scopus) | Journal’s CiteScore at time of analysis |
|  | Impact Factor (JCR 2023) | Journal Impact Factor according to JCR 2023 |
|  | Open Access | Whether the article is freely accessible (Yes/No) |
| Abstract characteristics | Abstract word limit | Word limit set by the journal for the abstract |
|  | Abstract word count – Journal | Word count reported by the journal |
|  | Abstract word count – Paper | Actual word count of the abstract in the paper |
|  | Compliance with word limit | Whether the abstract followed the journal’s limit (Yes/No) |
|  | Structured abstract | Presence of a structured abstract (Yes/No) |
| Methodological design | No. of primary outcomes | Number of primary outcomes assessed |
|  | No. of secondary outcomes | Number of secondary outcomes assessed |
|  | Distinction between primary and secondary outcomes | Whether primary and secondary outcomes were clearly defined (Yes/No) |
|  | Risk of bias assessment | Whether risk of bias was assessed (Yes/No) |
|  | Risk of bias tool | Tool used to assess bias (e.g., Cochrane, ROB-2) |
|  | GRADE assessment | Whether GRADE was used to evaluate certainty of evidence (Yes/No) |
| Reporting quality | Journal endorsement of ICMJE | Journal endorses ICMJE recommendations (Yes/No) |
|  | Journal endorsement of PRISMA | Journal endorses PRISMA guidelines (Yes/No) |
|  | Manuscript cites PRISMA | PRISMA cited in the manuscript (Yes/No) |
|  | Funding source reported | Whether the review disclosed a funding source (Yes/No) |
|  | Conflict of interest declared | Whether conflict of interest was reported (Yes/No) |
| Protocol registration | Protocol registered | Presence of registered protocol (Yes/No) |
|  | Registration platform | Registry used (e.g., PROSPERO, OSF, CDSR) |
|  | Protocol record type | Type of registration (prospective/retrospective) |
|  | Registration number | Identifier number in the registry |
| Spin-related variables | Presence of spin (abstract) | Whether spin was identified in the abstract (Yes/No) |
|  | Presence of spin (full text) | Whether spin was identified in the full text (Yes/No) |
|  | Type of spin | Category according to Yavchitz et al. (2016) – misleading reporting, interpretation, extrapolation |
|  | Level of spin | None, Low, or High according to severity |
|  | No. of spin identified | Count of individual spin strategies per review |
| Citation and visibility | Citation rate per year | Number of citations per year in Scopus |
|  | Total Scopus citations | Total number of citations in Scopus |
|  | No. of primary studies included | Number of primary studies included in the meta-analysis |
